# Altered cortical neuronal activity in functional esophageal disorders and its associations with chronic insomnia and peripheral inflammation: a resting-state fMRI study

**DOI:** 10.3389/fnmol.2026.1871404

**Published:** 2026-07-06

**Authors:** Bei Peng, Likun Zhong, Jinfu Huang, Meng Cui, Dan Huang, Yinfei Ouyang, Xiaoli Liang, Haoqiang Li, Xinghua Huang, Zhiming Lin, Yueming Liang, Chunling Huang, Yunxiao Liang, Demao Deng

**Affiliations:** 1 Department of Radiology, The People’s Hospital of Guangxi Zhuang Autonomous Region, Guangxi Academy of Medical Sciences, Nanning, China; 2 Department of Gastroenterology, The People’s Hospital of Guangxi Zhuang Autonomous Region, Nanning, China

**Keywords:** amplitude of low-frequency fluctuation, chronic insomnia disorder, disorders of gut-brain interaction (DBGIs), functional esophageal disorders, inflammation, middle temporal gyrus, resting-state fMRI

## Abstract

**Background/Objectives:**

Functional esophageal disorders (FEDs) are disorders of gut–brain interaction frequently accompanied by chronic insomnia disorder (CID), but their cortical neuronal basis remains unclear. This study aimed to investigate spontaneous cortical activity in FEDs using resting-state functional magnetic resonance imaging with amplitude of low-frequency fluctuation (ALFF) analysis and to examine its associations with sleep disturbance and peripheral inflammation.

**Methods:**

Nineteen patients with FEDs, 19 with CID, and 19 healthy controls (HCs) underwent resting-state fMRI and Pittsburgh Sleep Quality Index assessment. Patients with FEDs additionally completed symptom and psychological evaluations and underwent cytokine testing. Whole-brain ALFF was compared among groups, followed by FDR-corrected correlation analyses. An exploratory mediation model was presented in the Supplementary material for hypothesis generation only.

**Results:**

Compared with HCs, patients with FEDs showed reduced ALFF in the bilateral middle temporal gyri (MTG), right middle occipital gyrus, right precuneus, left postcentral gyrus, and left inferior parietal lobule. Compared with CID, FEDs showed lower ALFF in the bilateral MTG and left superior parietal gyrus. However, because MTG abnormalities have also been reported in chronic insomnia, this finding should not be interpreted as evidence that MTG reduction is exclusive to FEDs. In FEDs, nominally positive correlations were observed between left MTG ALFF and IL-8/TNF-*α*, and between right MTG ALFF and sleep medication use. An exploratory supplementary mediation model generated a hypothesis regarding a possible indirect association among IL-8, left MTG ALFF, and daytime dysfunction; however, this finding should not be interpreted causally. Across all participants, global and bilateral MTG ALFF values were negatively correlated with PSQI scores after FDR correction.

**Conclusion:**

FEDs showed reduced spontaneous cortical activity centered on the bilateral MTG, a region that may represent a transdiagnostic sleep- and sensory-related cortical hub rather than an FED-specific marker. This study provides new insights into the central mechanisms of FED and identifies candidate cortical regions for future mechanistic studies. These findings require validation in larger, adequately powered studies.

## 1 Introduction

Functional esophageal disorders (FEDs) are an important subtype of disorders of gut–brain interaction, including functional heartburn, functional chest pain, and reflux hypersensitivity (Schmulson, 2017; Aziz et al., 2016). These disorders present with persistent esophageal symptoms despite no structural abnormalities (Moudgal et al., 2021). Although visceral hypersensitivity is widely considered a central mechanism in FEDs, symptom generation is not likely to depend on peripheral sensitivity alone (Goyal et al., 2025). The perception of esophageal discomfort is shaped by central processing of afferent input, emotional arousal, symptom-related expectation, and cognitive interpretation (Gershon and Margolis, 2021). Esophageal hypervigilance and symptom-specific anxiety are important cognitive-affective constructs in chronic esophageal disease, including functional esophageal disorders. The Esophageal Hypervigilance and Anxiety Scale (EHAS) is a validated 15-item measure developed to assess these constructs in chronic esophageal conditions (Taft et al., 2018). Increasing evidence from studies on disorders of gut–brain interaction suggests that chronic gastrointestinal symptoms are linked to altered brain structure and function (Di Napoli et al., 2024), particularly in regions involved in pain modulation, self-referential processing, sensory integration, and salience attribution (Wan et al., 2025; Chen et al., 2021). The MTG is involved in multimodal sensory integration and semantic-affective processing, suggesting that MTG abnormalities may reflect altered integration of symptom-related sensory information rather than a purely esophagus-specific neural signature (Xu et al., 2022). However, compared with other gut-brain interaction disorders, the cortical functional characteristics of FEDs remain poorly understood (Hu et al., 2024).

Another important issue is the high frequency of sleep disturbance in patients with FEDs (Koloski et al., 2021). Chronic insomnia disorder (CID) frequently co-occurs with FEDs and may aggravate symptom perception, emotional distress, and daytime impairment (Wang et al., 2025). Poor sleep alters pain sensitivity, autonomic balance, inflammatory status, and resting-state brain activity (Riemann et al., 2020). Therefore, cortical abnormalities observed in FEDs likely partly reflect the effect of sleep disturbance rather than FED-related neural alterations alone (Koloski et al., 2021). In addition, peripheral inflammatory signaling may represent another biological link between esophageal symptoms and brain dysfunction (Gershon and Margolis, 2021). Cytokines can influence neuronal activity through humoral, autonomic, and vagal pathways (Thomas et al., 2011), whereas altered central regulation may, in turn, modulate immune function (Dressle et al., 2022). Consequently, investigating both sleep and inflammation is crucial for understanding how FEDs are embedded within a broader brain–body network rather than being limited to the esophagus itself.

Resting-state functional magnetic resonance imaging (rs-fMRI) is a useful approach for studying intrinsic brain activity, and amplitude of low-frequency fluctuation (ALFF) is a widely used index of spontaneous regional neuronal activity (Lv et al., 2018). In the present study, we compared ALFF among patients with FEDs, patients with CID, and healthy controls (HCs) to identify FED-related cortical activity changes and to compare FED-related cortical changes with those observed in a CID clinical control group. We further examined the correlations of abnormal ALFF values with sleep quality and peripheral inflammatory markers and performed an exploratory supplementary mediation analysis to generate hypotheses regarding possible indirect associations among inflammation, cortical activity, and sleep disturbance.

Accordingly, we hypothesized that: (i) FEDs would be associated with reduced spontaneous cortical activity in brain regions associated with visceral sensation and symptom integration, (ii) some abnormalities would show additional reduction in FEDs relative to CID, and (iii) key abnormal regions would show exploratory associations with sleep disturbance and inflammatory signaling.

## 2 Materials and methods

### 2.1 Participants

A total of 57 participants were enrolled, including 19 patients with FEDs, 19 patients with CID, and 19 HCs. The three groups were matched for age, sex, and educational level. All participants were right-handed adults aged 18–70 years. Written informed consent was obtained from each participant in accordance with the Declaration of Helsinki, and the study was approved by the ethics committee of the People’s Hospital of Guangxi Zhuang Autonomous Region. All participants were informed of their right to withdraw at any time without penalty.

**Figure 1:**
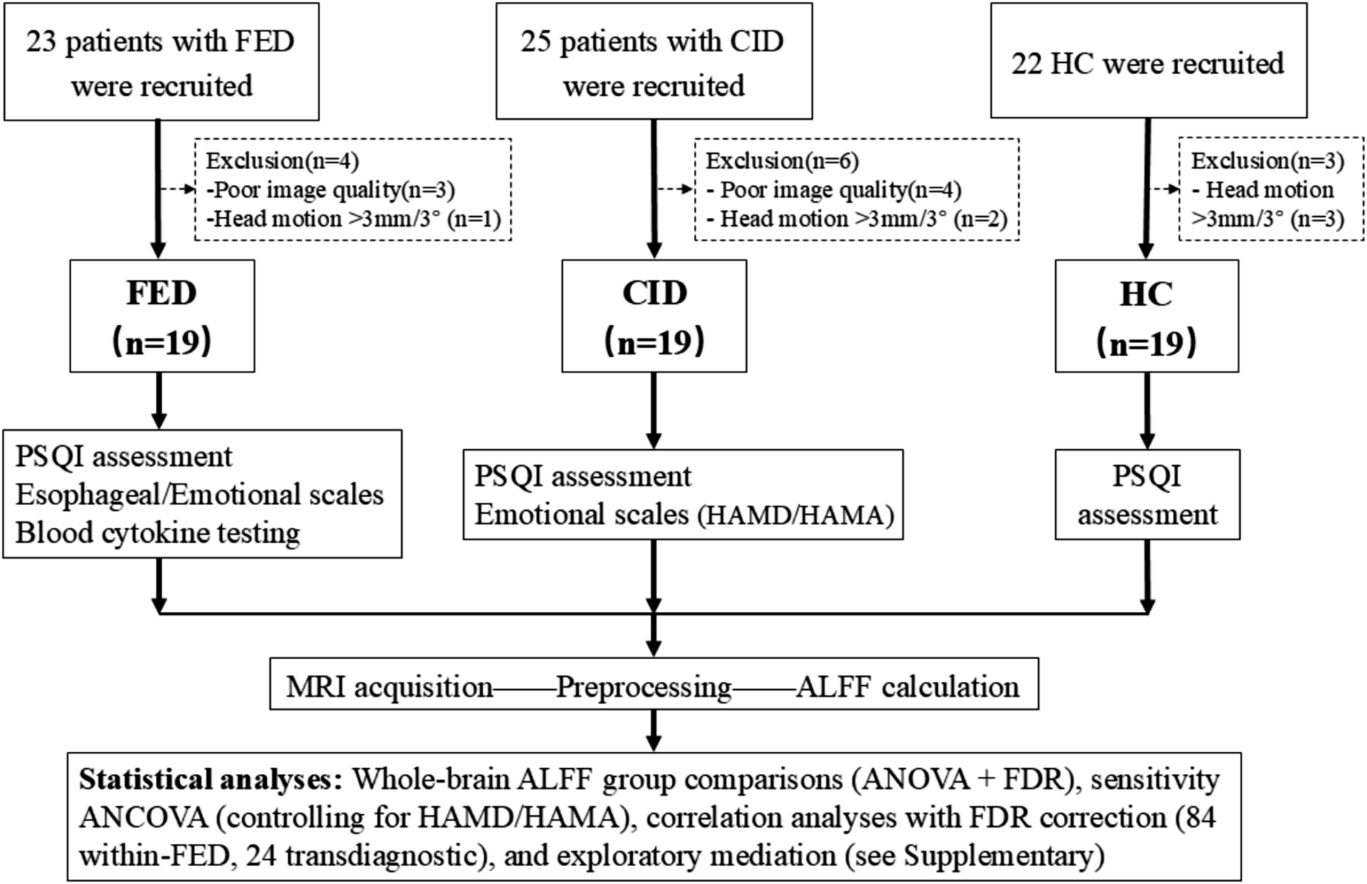
Study flowchart showing participant recruitment, clinical assessments, MRI acquisition, ALFF calculation, and statistical analyses (group comparisons, sensitivity ANCOVA, FDR corrected correlations, and exploratory mediation in Supplementary). FED, functional esophageal disorders; CID, chronic insomnia disorder; HC, healthy controls; ALFF, amplitude of low frequency fluctuation.

Patients with FEDs were recruited from the gastroenterology outpatient clinic and inpatient service, whereas patients with CID were recruited from a sleep clinic. HCs were recruited as volunteers without chronic insomnia symptoms or known digestive disease. A CID control group was included because sleep disturbance is common in FEDs and comparison with HCs alone would not adequately separate FED-associated neural alterations from sleep-related effects. Figure 1 shows an overview of the data collection and experimental procedures.

### 2.2 Diagnostic criteria

Patients with FEDs met the diagnostic criteria for FEDs—namely functional heartburn, functional chest pain, and reflux hypersensitivity—and had a history of heartburn or chest pain for at least 6 months, with fulfillment of the relevant diagnostic criteria during the previous 3 months. To ensure diagnostic specificity, all patients with FEDs underwent upper endoscopy with biopsy, high-resolution esophageal manometry, and 24-h pH monitoring and impedance testing. These tests were used to exclude eosinophilic esophagitis, major esophageal motility disorders, and gastroesophageal reflux disease, which could otherwise fully account for the patients’ symptoms.

Patients with CID met established diagnostic criteria for CID according to DSM-5, ICSD-3, and ICD-11. Symptoms were required to occur at least three times per week and persist for at least 3 months (Riemann et al., 2023). HCs had no known digestive disease or chronic insomnia symptoms.

### 2.3 Inclusion and exclusion criteria

The inclusion criteria were as follows: (1) age 18–70 years; (2) diagnosis of a FED for the FED group, CID for the CID group, or absence of digestive and sleep disorders for the HC group, all according to the abovementioned criteria; (3) right-handedness; (4) ability to discontinue proton pump inhibitors and prokinetic agents during the week before the study; and (5) when applicable, ability to skip insomnia medication on the night before imaging.

The exclusion criteria were as follows: (1) severe cardiopulmonary, neurological, or systemic disease preventing protocol completion; (2) major neurological or psychiatric disorders (e.g., stroke, brain tumor, or schizophrenia); (3) systemic diseases affecting gastrointestinal motility or sleep; (4) prior gastrointestinal, esophageal, or brain surgery; (5) for the FED group, the presence of structural digestive diseases (e.g., esophageal cancer, esophageal ulcer, reflux esophagitis, and major esophageal motility disorders); (6) for the CID group, the presence of other sleep disorders (e.g., moderate-to-severe sleep apnea or restless legs syndrome, and moderate-to-severe anxiety or depression); and (7) contraindications to MRI.

### 2.4 Clinical assessments

All participants completed the Pittsburgh Sleep Quality Index (PSQI), which was the measure of sleep quality in all groups. Both the PSQI total score and its subcomponents were used in subsequent analyses.

Notably, patients with FEDs underwent more detailed clinical phenotyping, including routine history taking and answering several validated questionnaires: Reflux Disease Questionnaire (RDQ), Esophageal Hypervigilance and Anxiety Scale (EHAS), Northwestern Esophageal Quality of Life scale (NEQOL), Patient Health Questionnaire-9 (PHQ-9), Generalized Anxiety Disorder-7 (GAD-7), Hamilton Depression Rating Scale (HAMD), and Hamilton Anxiety Rating Scale (HAMA). These measures were used to characterize symptom burden, hypervigilance, quality of life, and psychological status. The distributions of HAMD and HAMA scores in the FED group were examined for normality using the Shapiro–Wilk test. Non-parametric tests were applied when normality was violated. EHAS was administered only to the FED group because it was originally implemented as a disease-specific measure of esophageal symptom-related hypervigilance, and it was not included in the CID or HC assessment battery in the original study design.

After confirmation of eligibility, venous blood samples were collected from patients with FEDs for inflammatory cytokine testing. Serum samples were stored at 4 °C and sent for testing immediately. Cytokine testing was performed uniformly by the hospital clinical laboratory according to the manufacturer’s instructions and the laboratory standard operating procedure. Cytokine concentrations were measured using a 12-plex cytokine detection kit based on flow fluorescence immunoassay (Twelve-Cytokine Detection Kit, Hunan Unimedical Technology Co., Ltd., Changsha, China; manufactured by Changsha Yiyibio Bioengineering Co., Ltd., Changsha, China) on a Beckman Coulter DxFLEX flow cytometer. The assay quantified IL-1β, IL-2, IL-4, IL-5, IL-6, IL-8, IL-10, IL-12p70, IL-17, IFN-*α*, IFN-*γ*, and TNF-α. In brief, cytokine-specific capture beads and biotin-labeled detection antibodies were used to form sandwich immune complexes, followed by streptavidin–phycoerythrin fluorescence detection using APC and PE channels. Cytokine concentrations were calculated from standard curves. According to the manufacturer, the lower limit of detection for all 12 cytokines was no greater than 2.44 pg./mL, and the intra-assay and inter-assay coefficients of variation were ≤15%. The detailed analytical ranges are provided in Supplementary Table S3. When laboratory reports provided specific numerical values below the lower limit of detection, these original reported values were entered directly into the research database without additional retrospective conversion or imputation by the investigators.

### 2.5 MRI acquisition

MRI data were acquired on a Siemens MAGNETOM Vida 3.0-T scanner (MAGNETOM Vida 3.0 T; Siemens, Munich, Germany) with a 64-channel head coil. Both structural and resting-state images were obtained.

Three-dimensional T1-weighted structural images were acquired using an MPRAGE sequence with the following parameters: repetition time (TR) = 2000 ms, echo time (TE) = 2.05 ms, field of view (FOV) = 256 × 256 mm, slice thickness = 1 mm, matrix = 256 × 256, flip angle = 9°, and voxel size = 1 × 1 × 1 mm.

Resting-state functional MRI was acquired using an echo-planar imaging sequence with simultaneous multi-slice technology. The parameters were as follows: TR = 2000 ms, TE = 30 ms, FOV = 220 × 220 mm, slice thickness = 2.5 mm, 66 slices, 240 time points, flip angle = 90°, and voxel size = 2.5 × 2.5 × 2.5 mm. During scanning, participants were instructed to remain still, keep their eyes closed, stay awake, avoid deliberate thinking, and relax.

### 2.6 Rs-fMRI preprocessing

Image preprocessing was performed using Statistical Parametric Mapping 12 (SPM12)^1^ and the Data Processing Assistant for Resting-State fMRI (DPABI (Data Processing and Analysis of Brain Imaging)^2^ toolbox on the MATLAB (MathWorks, Natick, MA, USA) platform. The first 10 time points were discarded to allow signal stabilization. The remaining images were preprocessed in the following sequence: slice-timing and head-motion correction, co-registration to structural T1 images, and DARTEL-based segmentation into gray matter, white matter, and cerebrospinal fluid. Nuisance covariates included six rigid-body head-motion parameters, white matter signal, cerebrospinal fluid signal, global mean signal, and linear trends. Spatial normalization was performed with a resampled voxel size of 3 × 3 × 3 mm^3^, followed by temporal filtering at 0.01–0.08 Hz. Participants with head translation >3 mm or rotation >3° were excluded.

### 2.7 ALFF calculation

ALFF was calculated using DPABI. For each voxel, the preprocessed time series was transformed into the frequency domain using a fast Fourier transform. Then, the square root of the power spectrum within the low-frequency range of 0.01–0.08 Hz was computed to generate whole-brain ALFF maps. ALFF was used as an index of spontaneous local neuronal activity, with lower and higher values indicating reduced intrinsic activity and increased spontaneous activity, respectively. As a complementary sensitivity analysis, fractional ALFF (fALFF) was also calculated as the ratio of low-frequency power within 0.01–0.08 Hz to the total power across the detectable frequency range. fALFF maps were analyzed using the same group-comparison framework and FDR correction thresholds as the primary ALFF analysis.

### 2.8 Statistical analysis

Demographic and clinical variables (sex, age, education, PSQI, HAMD, HAMA) were compared among the three groups. For continuous variables, one-way analysis of variance (ANOVA) was used when data were normally distributed; otherwise, the Kruskal-Wallis test was applied. Post-hoc pairwise comparisons were performed with Bonferroni correction. For the comparison of HAMD and HAMA scores between the FED and CID groups, the Mann–Whitney U test was used because the FED group data deviated from normality (Shapiro–Wilk, both *p <* 0.05). Categorical variables were compared using the chi-square test.

#### 2.8.1 ALFF group comparisons

Whole-brain ALFF differences among the three groups were assessed using one-way ANOVA, followed by independent-samples t tests for the HC vs. FED and CID vs. FED comparisons. False discovery rate (FDR) correction was applied at the cluster level (voxel-level *p <* 0.001, cluster-level *p <* 0.05).

To control for potential confounding effects of psychological distress, we further compared FED and CID for the key extracted ALFF measures showing significant group differences, including left MTG, right MTG, and global ALFF, using analysis of covariance (ANCOVA) with HAMD, HAMA, age, sex, and the group-by-sex interaction as covariates. The significance threshold was set at *p <* 0.05 after FDR correction for the three regions. Partial eta-squared ( $\eta_{p}^{2}$ ) was calculated as a measure of effect size.

#### 2.8.2 Correlation analyses within the FED group (*n =* 19)

Spearman’s rank correlations were computed between ALFF values (global, left MTG, right MTG) and all clinical and inflammatory variables (84 tests). To correct for multiple comparisons, FDR correction (Benjamini–Hochberg) was applied across all 84 tests. Corrected *q* < 0.05 was considered statistically significant. Uncorrected *p*-values and FDR-corrected q values are both reported in Table 1, and the full matrix is provided in Supplementary Table S1. No correlation remained significant after FDR correction; therefore, no mediation analysis was performed in the main manuscript (exploratory mediation results are presented in the Supplementary material).

**Table 1:** Exploratory correlations between ALFF measures and clinical/inflammatory variables in the FED group (*n =* 19).

ALFF measure	Variable	Spearman’s r	Uncorrected *P*	FDR-corrected q
Global ALFF	IL-8	0.567	0.011	0.337
Global ALFF	TNF-α	0.518	0.023	0.386
Left MTG ALFF	IL-8	0.675	0.002	0.129
Left MTG ALFF	TNF-α	0.563	0.012	0.337
Right MTG ALFF	PSQI-F	0.541	0.017	0.354

MTG, middle temporal gyrus; PSQI-F, Pittsburgh Sleep Quality Index Component F (use of sleep medication). FDR correction was performed using the Benjamini–Hochberg method across all 84 correlation tests. FDR-corrected q values are shown for the nominal correlations; none remained statistically significant after correction. These correlations are presented as hypothesis-generating observations that require independent replication. The full correlation matrix is available in Supplementary Table S1.

#### 2.8.3 Transdiagnostic correlations (all participants, *N =* 57)

**Figure 2:**
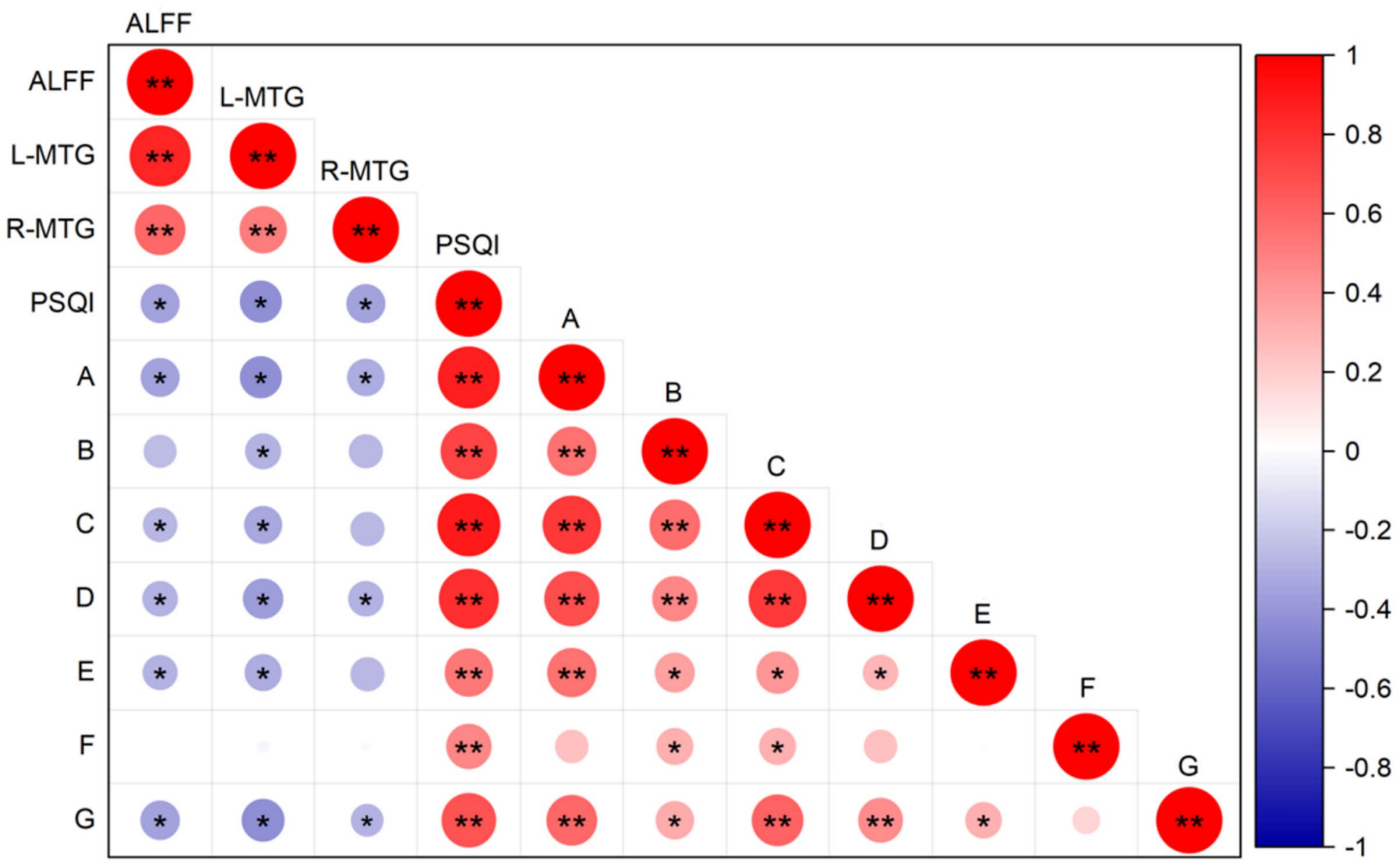
Correlations between ALFF values and sleep quality across all participants. After false discovery rate (FDR) correction for 24 comparisons (Benjamini–Hochberg, *q* < 0.05), the following correlations remained significant. Global ALFF was significantly negatively correlated with PSQI total score and components A, C, D, E, and G, whereas L-MTG ALFF was significantly negatively associated with the PSQI total score and components A, B, C, D, E, and G, and R-MTG was significantly negatively associated with the PSQI total score and components A, D, and G.

Correlations between ALFF measures (global, left MTG, right MTG) and PSQI scores (total score and seven subcomponents, 24 tests) were examined using Spearman’s rank correlation. FDR correction (Benjamini–Hochberg) was applied across all 24 tests. Correlations with *q* < 0.05 are reported in Table 2 and visualized in Figure 2.

**Table 2:** Significant correlations between ALFF measures and sleep quality across all participants (*N =* 57) after FDR correction.

ALFF measure	PSQI component	Spearman’s r	Uncorrected *P*	FDR-corrected q
Global ALFF	A (subjective sleep quality)	−0.374	0.0042	0.020
	C (sleep duration)	−0.295	0.0258	0.045
	D (sleep efficiency)	−0.285	0.0316	0.046
	E (sleep disturbances)	−0.306	0.0206	0.045
	G (daytime dysfunction)	−0.352	0.0072	0.029
	PSQI total score	−0.336	0.0106	0.036
Left MTG	A (subjective sleep quality)	−0.452	0.00041	0.010
	B (sleep latency)	−0.284	0.0325	0.046
	C (sleep duration)	−0.312	0.0181	0.043
	D (sleep efficiency)	−0.381	0.0035	0.020
	E (sleep disturbances)	−0.299	0.0238	0.045
	G (daytime dysfunction)	−0.397	0.0022	0.018
	PSQI total score	−0.402	0.0019	0.018
Right MTG	A (subjective sleep quality)	−0.321	0.0149	0.040
	D (sleep efficiency)	−0.329	0.0123	0.037
	G (daytime dysfunction)	−0.290	0.0286	0.046
	PSQI total score	−0.295	0.0259	0.045

MTG, middle temporal gyrus; PSQI, Pittsburgh Sleep Quality Index. FDR correction was performed using the Benjamini–Hochberg method across 24 tests (3 ALFF measures × 8 PSQI scores). All *q*-values listed are < 0.05 after monotonicity adjustment. Only correlations with FDR-corrected *q* < 0.05 are shown. For the full correlation matrix including non-significant results (see Supplementary Table S2).

No formal *a priori* power calculation was performed because this was an exploratory rs-fMRI study involving rigorously phenotyped FED patients. The sample size was expected to detect only relatively large group-level effects and was not intended to provide stable estimates for brain–behavior, brain–inflammation, or mediation analyses. Therefore, all correlation and mediation analyses were treated as exploratory and hypothesis-generating. All statistical tests were two-tailed. Data are presented as mean ± standard deviation, median (interquartile range), or frequency. Analyses were performed using SPSS version 25.0 and R version 4.0.

## 3 Results

### 3.1 Demographic and clinical characteristics

Demographic and clinical characteristics are shown in Table 3. The FED, CID, and HC groups did not differ significantly in terms of sex distribution, age, and educational level, indicating adequate matching across groups. Conversely, PSQI scores differed significantly among groups. The mean PSQI scores in the FED, CID, and HC groups were 12.95 ± 4.30, 13.11 ± 3.23, and 2.89 ± 1.45, respectively (*p <* 0.001). Although *post-hoc* analysis revealed no significant difference between the FED and CID groups (*p* = 0.952), both patient groups had significantly poorer sleep quality than HCs, supporting the inclusion of patients with CID as a disease control group.

**Table 3:** Demographic and clinical characteristics of the FED, CID, and HC groups.

Variable	FED (*n =* 19)	CID (*n =* 19)	HC (*n =* 19)	Statistic	*P-*value
Sex (female/male)	12/7	12/7	12/7	*χ*^2^ < 0.001	>0.999
Age (years)	54.05 ± 9.97	53.95 ± 8.57	54.11 ± 6.91	*F* = 0.002	0.998
Education (years)	11.00 ± 3.50	12.42 ± 4.23	10.84 ± 3.17	*F* = 1.073	0.349
PSQI total score	12.95 ± 4.30	13.11 ± 3.23	2.89 ± 1.45	*F* = 62.866	<0.001
HAMD score	10 (7–21)	7 (6–8)	–	U = 125.5	0.107
HAMA score	8 (6–14)	6 (4–7)	–	U = 96.0	**0.013**

Data are presented as mean ± standard deviation, median (interquartile range), or frequency. FED, functional esophageal disorders; CID, chronic insomnia disorder; HC, healthy controls; PSQI, Pittsburgh Sleep Quality Index; HAMD, Hamilton Depression Rating Scale; HAMA, Hamilton Anxiety Rating Scale. *χ*^2^: Chi-square statistic; F: F-statistic from one-way ANOVA; U: Mann–Whitney U statistic. The HAMD and HAMA scores in the FED group were not normally distributed (Shapiro–Wilk test, both *p <* 0.05); therefore, median (IQR) is reported and the Mann–Whitney U test was used for group comparisons (FED vs. CID). The HC group did not complete HAMD/HAMA assessments; their eligibility was confirmed by medical history, clinical interview, and the absence of sleep or digestive disorders, as described in Section 2.2 and 2.3. Bold *P*-value indicates statistical significance (*p <* 0.05).

### 3.2 Whole-brain ALFF abnormalities in FED and sensitivity analysis

**Figure 3:**
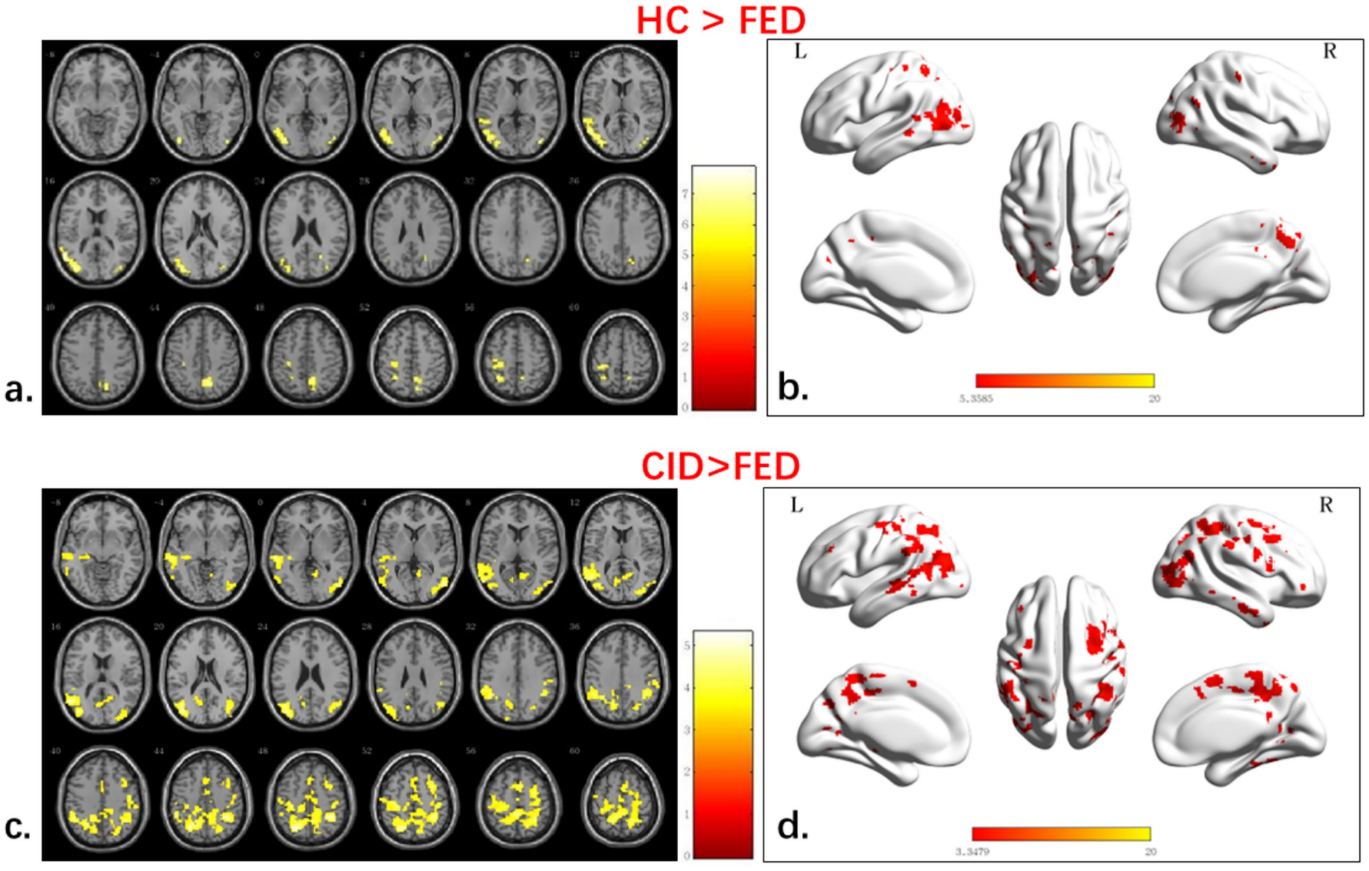
Brain regions showing significantly lower ALFF in patients with functional esophageal disorders (FEDs) than in healthy controls (HCs) and patients with chronic insomnia disorder (CID). **(a,b)** Compared with HCs, patients with FEDs showed reduced ALFF in the bilateral middle temporal gyri, right middle occipital gyrus, right precuneus, left postcentral gyrus, and left inferior parietal lobule. **(c,d)** Compared with patients having CID, those with FEDs showed reduced ALFF in the bilateral middle temporal gyri and left superior parietal gyrus.

Compared with HCs, patients with FEDs showed significantly reduced ALFF in multiple cortical regions, including the bilateral middle temporal gyri (MTG), right middle occipital gyrus, right precuneus, left postcentral gyrus, and left inferior parietal lobule (Table 4 and Figure 3). These findings suggest that FEDs are associated with widespread reductions in spontaneous cortical activity involving temporal, parietal, somatosensory, and occipital regions.

**Table 4:** Brain regions showing significant ALFF differences in HC > FED and CID > FED comparisons.

Brain regions	Cluster size	MNI coordinates			Peak *t*-value
		X	Y	Z	
HC > FED					
Right middle temporal region	59	42	18	−39	6.6497
Left middle temporal gyrus	363	−54	−60	15	7.8488
Right middle occipital gyrus	59	39	−84	6	6.8832
Right precuneus	157	12	−63	45	6.9879
Left postcentral gyrus	82	−27	−30	54	6.4809
Left inferior parietal lobule	44	−30	−54	54	6.8051
CID > FED					
Left middle temporal gyrus	766	−54	−60	15	5.2360
Right middle temporal gyrus	314	54	−72	3	4.7941
Left superior parietal gyrus	2,873	−27	−66	48	5.3286

FED, functional esophageal disorders; CID, chronic insomnia disorder; HC, healthy controls. FDR-corrected, cluster-level: *P <* 0.05, voxel-level: *p <* 0.001.

**Figure 4:**
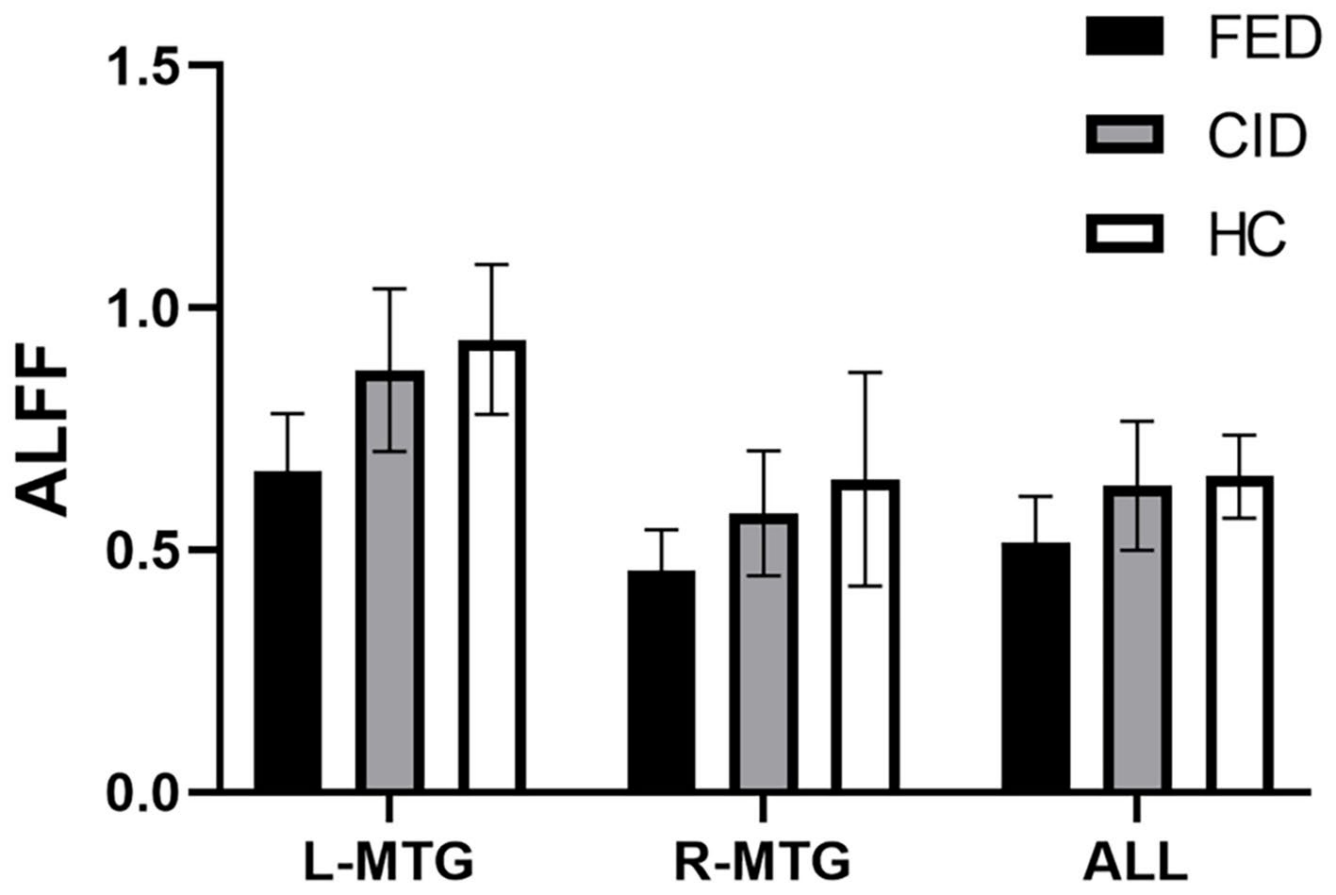
Group comparisons of global ALFF, left middle temporal gyrus ALFF, and right middle temporal gyrus ALFF among the FED, CID, and HC groups.

When patients with FEDs were compared directly with those having CID, the former showed significantly reduced ALFF in the bilateral MTG and left superior parietal gyrus (Table 4 and Figure 3). Because MTG abnormalities have also been reported in chronic insomnia, this finding should not be interpreted as indicating that MTG reduction is exclusive to FEDs. Rather, the lower ALFF observed in FEDs relative to CID suggests additional FED-related modulation of a transdiagnostic sleep- and sensory-related cortical region. Consistently, global ALFF as well as the left and right MTG ALFF values were all lower in the FED group than in the other two groups (Figure 4).

### 3.3 Sensitivity analysis: controlling for psychological confounders

To determine whether the observed ALFF differences between FED and CID persisted after adjustment for anxiety and depression, we performed an analysis of covariance (ANCOVA) for the regions that showed significant group differences (left MTG, right MTG, and global ALFF). The model included HAMD and HAMA scores as covariates, while also controlling for age, sex, and the group-by-sex interaction.

After adjustment, the group effect remained significant for all three regions. Table 5 summarises the ANCOVA results. In brief, the left MTG showed a significant group difference [*F*(1,31) = 17.07, *p* = 0.00025, partial *η*^2^ = 0.355], followed by the right MTG [*F*(1,31) = 8.61, *p* = 0.0063, partial *η*^2^ = 0.217] and global ALFF [*F*(1,31) = 5.61, *p* = 0.024, partial *η*^2^ = 0.153]. After false discovery rate (FDR) correction for the three comparisons, all regions remained significant (left MTG: *q =* 0.0008; right MTG: *q =* 0.009; global ALFF: *q =* 0.024). These results indicate that the reduced spontaneous cortical activity in FED patients compared with CID patients is not fully explained by group differences in anxiety or depression levels. The bilateral MTG and global ALFF reductions remained significant after adjustment for psychological distress.

**Table 5:** ANCOVA results for FED vs. CID comparisons after controlling for HAMD, HAMA, age, sex, and group-by-sex interaction.

Brain region	*F* (df)	*P*	Partial *η*^2^	FDR-corrected *q*
Left MTG	17.07 (1, 31)	0.001	0.355	0.001
Right MTG	8.61 (1, 31)	0.006	0.217	0.009
Global ALFF	5.61 (1, 31)	0.024	0.153	0.024

MTG, middle temporal gyrus; ALFF, amplitude of low-frequency fluctuation. ANCOVA included HAMD, HAMA, age, sex, and group-by-sex interaction as covariates. FDR correction was applied across the three regions (Benjamini–Hochberg). PP-values and FDR-corrected qq-values are rounded to three decimal places; PP-values less than 0.001 are reported as < 0.001<0.001. Bold values indicate statistical significance (*p* < 0.05 *p*<0.05).

### 3.4 Correlation analysis within the FED group

We examined Spearman’s rank correlations between ALFF measures (global ALFF, left MTG ALFF, right MTG ALFF) and clinical/inflammatory variables in the FED group (*n =* 19). After false discovery rate (FDR) correction for 84 comparisons (Benjamini–Hochberg, *q* < 0.05), no correlation remained statistically significant (all *q* ≥ 0.129).

**Table** **1** lists the nominal correlations with uncorrected *p <* 0.05. These included global ALFF with IL-8 (r = 0.567, uncorrected *p* = 0.011) and TNF-*α* (r = 0.518, uncorrected *p* = 0.023); left MTG ALFF with IL-8 (r = 0.675, uncorrected *p* = 0.002) and TNF-α (r = 0.563, uncorrected *p* = 0.012); and right MTG ALFF with PSQI-F (use of sleep medication, r = 0.541, uncorrected *p* = 0.017). All other correlations were not significant even at the uncorrected level.

Thus, no formal mediation analysis was performed in the main manuscript. An exploratory mediation model (based on the nominal IL-8 – left MTG ALFF – daytime dysfunction pathway) is presented in the Supplementary Results and Supplementary Figure S2. An exploratory correlation plot for the nominal correlations is provided in Supplementary Figure S1, and the full correlation matrix (all 84 tests) is available in Supplementary Table S1.

### 3.5 Correlations between ALFF and sleep quality across all participants

To examine transdiagnostic associations between spontaneous cortical activity and sleep quality, we performed Spearman’s rank correlation analyses between ALFF values (global, left MTG, right MTG) and PSQI scores (total score and seven subcomponents) in the combined sample of all 57 participants (FED, CID, and HC groups). After false discovery rate (FDR) correction for 24 multiple comparisons (Benjamini–Hochberg, *q* < 0.05), 17 correlations remained statistically significant, as summarised in Table 2.

Global ALFF showed significant negative correlations with the PSQI total score (r = −0.336, *q =* 0.036), as well as with subcomponents A (subjective sleep quality, r = −0.374, *q =* 0.020), C (sleep duration, r = −0.295, *q =* 0.045), D (sleep efficiency, r = −0.285, *q =* 0.046), E (sleep disturbances, r = −0.306, *q =* 0.045), and G (daytime dysfunction, r = −0.352, *q =* 0.029). Correlations with PSQI-B (sleep latency) and PSQI-F (use of sleep medication) did not survive FDR correction.

Left MTG ALFF exhibited the broadest pattern of significant associations, correlating negatively with the PSQI total score (r = −0.402, *q =* 0.018) and all subcomponents except PSQI-F: A (r = −0.452, *q =* 0.010), B (r = −0.284, *q =* 0.046), C (r = −0.312, *q =* 0.043), D (r = −0.381, *q =* 0.020), E (r = −0.299, *q =* 0.045), and G (r = −0.397, *q =* 0.018).

Right MTG ALFF was significantly correlated with the PSQI total score (r = −0.295, *q =* 0.045) and with subcomponents A (r = −0.321, *q =* 0.040), D (r = −0.329, *q =* 0.037), and G (r = −0.290, *q =* 0.046). Its correlations with PSQI-B, PSQI-C, PSQI-E, and PSQI-F were not significant after correction.

Taken together, lower spontaneous cortical activity, particularly in the left MTG, was associated with poorer sleep quality across diagnostic categories. These findings support a transdiagnostic association between reduced ALFF and sleep disturbance.

## 4 Discussion

The present study investigated spontaneous cortical neuronal activity in FEDs using resting-state fMRI and ALFF analysis, with patients having CID included as a disease control group. Several findings merit emphasis. First, compared with HCs, patients with FEDs showed reduced ALFF in several cortical regions, including the bilateral MTG, right middle occipital gyrus, right precuneus, left postcentral gyrus, and left inferior parietal lobule. Second, compared with patients with CID, those with FEDs still showed significantly reduced ALFF in the bilateral MTG and left superior parietal gyrus. Third, the bilateral MTG showed additional reduction in FEDs relative to both HCs and the CID control group. Given prior evidence of MTG abnormalities in chronic insomnia, these findings should be interpreted as an FED-associated modulation in this cohort rather than evidence of a FED-specific MTG biomarker. After controlling for anxiety (HAMA) and depression (HAMD) using ANCOVA, the group differences in the bilateral MTG and global ALFF between FED and CID remained significant (all FDR-corrected *q* < 0.05), with corresponding partial *η*^2^ estimates reported in Table 5. All FED-group correlation and mediation findings were exploratory and should be interpreted with caution. Fourth, in exploratory analyses within the FED group, nominal positive correlations were observed between left MTG ALFF and IL-8/TNF-*α*, and between right MTG ALFF and sleep medication use; however, none survived FDR correction. Fifth, an exploratory supplementary mediation model generated a hypothesis regarding a possible indirect association of IL-8 on daytime dysfunction via left MTG ALFF, but this finding did not withstand multiple comparison correction and should be interpreted with caution (see Supplementary material).

A notable finding is the association of FEDs with a distributed pattern of reduced spontaneous cortical activity rather than a single focal abnormality. The affected regions involve temporal, parietal, somatosensory, and occipital cortices, indicating that FEDs should be interpreted and explored as disorders resulting from broader brain–gut dysregulation rather than purely peripheral esophageal diseases. The right precuneus and parietal regions are closely associated with default mode network processing, including self-referential cognition, internal monitoring, and integration of ongoing bodily experience (Raichle, 2015; Fiúza-Fernandes et al., 2025). Reduced ALFF in these areas may reflect altered processing of interoceptive signals or long-term adaptation to persistent symptom burden (Ray and Ghoshal, 2024; Verdonk and Khalsa, 2025). The left postcentral gyrus, as a part of the somatosensory network, may be relevant to abnormal bodily sensation and pain-related processing, particularly in patients with esophageal discomfort or chest pain (Croosu et al., 2023). The involvement of the right middle occipital gyrus may appear less intuitive; however, visual-associative cortices participate in anticipatory processing, symptom imagery, and multimodal integration, all of which can contribute to chronic symptom perception (Fiúza-Fernandes et al., 2025; Ding et al., 2025). Although the bilateral MTG was the most consistently abnormal region across comparisons, the involvement of the right middle occipital gyrus (visual-associative cortex), precuneus (default mode network), postcentral gyrus (somatosensory), and inferior parietal lobule (multimodal integration) highlights that FEDs affect multiple large-scale networks beyond the temporal lobe. Overall, these findings suggest widespread cortical functional alterations in FEDs.

A key finding of this study is the involvement of the bilateral MTG. These regions were abnormal in comparison with not only HCs but also patients with CID. Our MTG findings should be interpreted in the context of previous insomnia studies reporting temporal abnormalities, including bilateral MTG ALFF reductions (Wu et al., 2020). As the FED and CID groups had similarly impaired PSQI scores, the lower ALFF in the bilateral MTG in the FED group may not be fully attributable to insomnia or generalized fatigue. Instead, the MTG may represent an FED-associated, but not FED-exclusive, cortical alteration. The MTG is involved in multimodal sensory integration (Xu et al., 2022), semantic-affective processing (Pan et al., 2025), contextual memory (Lu et al., 2022), and pain-related cognition (Biggs et al., 2020). In chronic conditions, it may contribute more to the interpretation and persistence of bodily discomfort than to sensory input alone (Shinto et al., 2025). For patients with FED, repeated esophageal symptoms may induce long-term changes in how internal bodily sensations are integrated with expectation, attention, and emotional meaning. Therefore, reduced resting-state activity in this region may reflect altered central reorganization after chronic symptom-related over-engagement.

We observed nominal correlations suggesting a possible but unconfirmed asymmetry between the left and right MTG in the FED group, with left MTG ALFF showing uncorrected positive associations with IL-8 and TNF-*α*, and right MTG ALFF showing an uncorrected positive association with sleep medication use. The left MTG may be associated with immune–inflammatory signaling (Zhang et al., 2024). Peripheral cytokines such as IL-8 and TNF-α can signal the brain through several pathways: active transport across the blood–brain barrier, binding to endothelial receptors, and vagal afferent activation (Thomas et al., 2011; Biggs et al., 2020). Conversely, the right MTG may be related to sleep-related behavior (Shao et al., 2025), suggesting a possible role in subjective perception and behavioral management of sleep disturbance (Pan et al., 2025; Paulekiene et al., 2022). However, after FDR correction for 84 comparisons, none of these correlations remained statistically significant (all q ≥ 0.129). Therefore, any claim of a lateralized “left immune, right sleep” pattern would be premature. These exploratory observations should be interpreted with caution and require independent replication in larger cohorts.

An exploratory mediation model was examined in the Supplementary material, suggesting a possible indirect effect of IL-8 on daytime dysfunction via left MTG ALFF. However, because the underlying correlations did not survive FDR correction and the data are cross-sectional, this finding should be viewed as hypothesis-generating only and does not support causal claims of “buffering” or “compensation.”

Overall, group-nonspecific correlations between ALFF and the PSQI provide an additional perspective. Global ALFF and bilateral MTG ALFF were negatively correlated with the PSQI total score and several PSQI components, indicating that poorer sleep quality is linked to lower spontaneous cortical activity across diagnostic categories (Riemann et al., 2020). In FEDs, sleep disturbance may further exacerbate FED-related neural alterations. The associations of a broad set of PSQI dimensions—including subjective sleep quality, sleep latency, sleep duration, sleep efficiency, sleep disturbances, and daytime dysfunction—with the left MTG ALFF further underscore the relevance of this region to the interaction between sleep disruption and symptom-related brain dysfunction.

These findings may have several clinical implications. First, the bilateral MTG may represent candidate regions for future validation studies examining how FED-related symptom processing interacts with transdiagnostic sleep-related cortical alterations. Second, although the lateralized correlations did not survive multiple comparison correction, the observed nominal patterns generate hypotheses for future studies. For example, patients with prominent inflammatory burden, fatigue, or daytime dysfunction may be more strongly linked to left temporal mechanisms (Zhang et al., 2024; Du et al., 2022); in contrast, patients with marked sleep-related behavioral disturbances may be more closely associated with right temporal dysfunction (Paulekiene et al., 2022). These possibilities need to be tested in confirmatory studies with larger samples. Third, the findings reinforce the importance of identifying and treating sleep disturbance in patients with FED—even when insomnia is not the primary complaint—as sleep-related cortical changes may accompany symptom exacerbation.

This study has several limitations. First, the cross-sectional design precludes causal inference; longitudinal or interventional studies are warranted to clarify the temporal relationships among inflammation, cortical activity, and clinical symptoms. Second, the sample size was modest and derived from a single center, which limits generalizability and may reduce statistical power, particularly in correlation analyses. Given the modest sample size, the observed effect sizes, particularly for brain–behavior and brain–inflammation associations, may be inflated due to the so-called winner’s curse; therefore, these estimates should be interpreted cautiously and require independent replication. No formal *a priori* power calculation was performed; therefore, this study should be viewed as a preliminary neuroimaging investigation rather than a definitive biomarker study. Third, although ALFF reflects regional spontaneous activity, it does not capture functional connectivity or network-level organization. Future studies should incorporate resting-state functional connectivity (rsFC) analyses and multimodal imaging to clarify how the MTG interacts with other key regions in visceral and sleep-related processing. Fourth, FEDs represent a heterogeneous category, and the current sample size precluded subgroup analysis of functional heartburn, functional chest pain, and reflux hypersensitivity. Future multi-center studies with larger samples are needed to address this question. Fifth, esophageal hypervigilance was assessed only in the FED group using the EHAS. This reflected the original study design, in which EHAS was used as a disease-specific measure of esophageal symptom-related hypervigilance rather than as a transdiagnostic sensory hypervigilance scale. However, this design limits our ability to determine whether the observed MTG differences are independent of general sensory hypervigilance or disease-specific attentional bias toward esophageal sensations. Future studies should assess esophageal and broader sensory hypervigilance across all groups. Sixth, we additionally performed fractional ALFF (fALFF) analyses as a complementary measure of spontaneous brain activity. However, after applying the same FDR correction for multiple comparisons (both for group differences and correlations), no significant group differences or correlations were observed for fALFF (Supplementary Table S4). The absence of significant fALFF findings suggests that the ALFF results may partly reflect differences in absolute low-frequency signal amplitude rather than frequency-normalized regional activity. Therefore, the ALFF findings should be interpreted cautiously and should not be regarded as definitive evidence of neuronal specificity. Future studies with larger samples and simultaneous physiological monitoring are needed to compare the sensitivity of ALFF and fALFF in FEDs.

## 5 Conclusion

FEDs showed reduced spontaneous cortical activity in the bilateral MTG and other cortical regions. Because MTG abnormalities have also been reported in chronic insomnia, MTG reduction should not be considered exclusive to FEDs. Exploratory correlation and mediation findings did not survive multiple comparison correction and require independent replication. The main group-level finding is that FED patients exhibit reduced ALFF in the bilateral MTG and other cortical regions compared with both HCs and CID patients in this cohort. This study provides new insights into the central mechanisms of FED and identifies candidate cortical regions for future mechanistic studies rather than definitive biomarkers.

## Data Availability

The original contributions presented in the study are included in the article/Supplementary material, further inquiries can be directed to the corresponding authors.

## Footnotes

1
https://www.fil.ion.ucl.ac.uk/spm/software/spm12/

2
http://rfmri.org/DPABI

## References

[ref1] Aziz Q. Fass R. Gyawali C. P. Miwa H. Pandolfino J. E. Zerbib F. (2016). Esophageal disorders. Gastroenterology 150, 1368–1379. doi: 10.1053/j.gastro.2016.02.012, 27144625

[ref2] Biggs E. E. Timmers I. Meulders A. Vlaeyen J. W. S. Goebel R. Kaas A. L. (2020). The neural correlates of pain-related fear: a meta-analysis comparing fear conditioning studies using painful and non-painful stimuli. Neurosci. Biobehav. Rev. 119, 52–65. doi: 10.1016/j.neubiorev.2020.09.016, 33011229

[ref3] Chen X.-F. Guo Y. Lu X.-Q. Qi L. Xu K. H. Chen Y. . (2021). Aberrant intraregional brain activity and functional connectivity in patients with diarrhea-predominant irritable bowel syndrome. Front. Neurosci. 15:721822. doi: 10.3389/fnins.2021.721822, 34539337 PMC8446353

[ref4] Croosu S. S. Røikjer J. Mørch C. D. Ejskjaer N. Frøkjær J. B. Hansen T. M. (2023). Alterations in functional connectivity of thalamus and primary somatosensory cortex in painful and painless diabetic peripheral neuropathy. Diabetes Care 46, 173–182. doi: 10.2337/dc22-0587, 36469731

[ref5] Di Napoli A. Pasquini L. Visconti E. Vaccaro M. Rossi-Espagnet M. C. Napolitano A. (2024). Gut-brain axis and neuroplasticity in health and disease: a systematic review. Radiol. Med. 130, 327–358. doi: 10.1007/s11547-024-01938-0, 39718685

[ref6] Ding J. Yu M. Li L. Yang M. Yang P. Hua B. . (2025). Aberrant intra-network resting-state functional connectivity in chronic insomnia with or without cognitive impairment. Neuroscience 565, 257–264. doi: 10.1016/j.neuroscience.2024.11.046, 39579856

[ref7] Dressle R. J. Feige B. Spiegelhalder K. Schmucker C. Benz F. Mey N. C. . (2022). HPA axis activity in patients with chronic insomnia: a systematic review and meta-analysis of case-control studies. Sleep Med. Rev. 62:101588. doi: 10.1016/j.smrv.2022.101588, 35091194

[ref8] Du Y. Y. Z W. Zhou X. L. Zeng M. Yang D. H. Xie X. Z. . (2022). Survivors of COVID-19 exhibit altered amplitudes of low frequency fluctuation in the brain: a resting-state functional magnetic resonance imaging study at 1-year follow-up. Neural Regen. Res. 17, 1576–1581. 34916443 10.4103/1673-5374.327361 PMC8771089

[ref9] Fiúza-Fernandes J. Pereira-Mendes J. Esteves M. Radua J. Picó-Pérez M. Leite-Almeida H. (2025). Common neural correlates of chronic pain – a systematic review and meta-analysis of resting-state fMRI studies. Prog. Neuro-Psychopharmacol. Biol. Psychiatry 138:111326. doi: 10.1016/j.pnpbp.2025.111326, 40086716

[ref10] Gershon M. D. Margolis K. G. (2021). The gut, its microbiome, and the brain: connections and communications. J. Clin. Invest. 131:e143768. doi: 10.1172/JCI143768, 34523615 PMC8439601

[ref11] Goyal O. Goyal P. Goyal M. K. Jain K. Midha V. Sood A. (2025). Overlap of ‘disorders of gut-brain interaction’ and their impact on quality of life and somatization in a tertiary care center- a cross-sectional study. Indian J. Gastroenterol. 44, 478–488. doi: 10.1007/s12664-025-01770-y, 40232666

[ref12] Hu Y. Zhao J. Jin Y. Du Y. Zhao Q. Xu S. . (2024). The altered resting-state functional connectivity of thalamic subregions in patients with globus pharyngeus. Brain Imaging Behav. 19, 23–31. doi: 10.1007/s11682-024-00940-4, 39417942

[ref13] Koloski N. A. Jones M. Walker M. M. Keely S. Holtmann G. Talley N. J. (2021). Sleep disturbances in the irritable bowel syndrome and functional dyspepsia are independent of psychological distress: a population-based study of 1322 Australians. Aliment. Pharmacol. Ther. 54, 627–636. doi: 10.1111/apt.16500, 34247414

[ref14] Lu F. Yang W. Wei D. Sun J. Zhang Q. Qiu J. (2022). Superior frontal gyrus and middle temporal gyrus connectivity mediates the relationship between neuroticism and thought suppression. Brain Imaging Behav. 16, 1400–1409. doi: 10.1007/s11682-021-00599-1, 35041138

[ref15] Lv H. Wang Z. Tong E. Williams L. M. Zaharchuk G. Zeineh M. . (2018). Resting-state functional MRI: everything that nonexperts have always wanted to know. AJNR Am. J. Neuroradiol. 39, 1390–1399. doi: 10.3174/ajnr.A5527, 29348136 PMC6051935

[ref16] Moudgal R. Schultz A. W. Shah E. D. (2021). Systemic disease associations with disorders of gut–brain interaction and gastrointestinal transit: a review. Clin. Exp. Gastroenterol. 14, 249–257. doi: 10.2147/CEG.S283685, 34135613 PMC8197439

[ref17] Pan N. C. Gao R. Ma K. Qiao L. Ni D. Yu T. . (2025). Left insula and right middle temporal gyrus dominate cortical network discriminating arousal-dependent emotions. Adv. Sci. 12:e2411790. doi: 10.1002/advs.202411790, 39823533 PMC11905088

[ref18] Paulekiene G. Pajarskiene M. Pajediene E. Radziunas A. (2022). Sleep dysfunction and Grey matter volume. Curr. Neurol. Neurosci. Rep. 22, 275–283. doi: 10.1007/s11910-022-01190-x, 35364772

[ref19] Raichle M. E. (2015). The brain's default mode network. Annu. Rev. Neurosci. 38, 433–447. doi: 10.1146/annurev-neuro-071013-014030, 25938726

[ref20] Ray G. Ghoshal U. C. (2024). Epidemiology of disorders of the gut-brain interaction: an appraisal of the Rome IV criteria and beyond. Gut and Liver. 18, 578–592. doi: 10.5009/gnl230396, 38680110 PMC11249947

[ref21] Riemann D. Espie C. A. Altena E. Arnardottir E. S. Baglioni C. Bassetti C. L. A. . (2023). The European insomnia guideline: an update on the diagnosis and treatment of insomnia 2023. J. Sleep Res. 32:e14035. doi: 10.1111/jsr.14035, 38016484

[ref22] Riemann D. Krone L. B. Wulff K. Nissen C. (2020). Sleep, insomnia, and depression. Neuropsychopharmacology 45, 74–89. doi: 10.1038/s41386-019-0411-y, 31071719 PMC6879516

[ref23] Schmulson M. J. D D. (2017). What is new in Rome IV. J. Neurogastroenterol. Motil. 23, 151–163. 28274109 10.5056/jnm16214 PMC5383110

[ref24] Shao Y. Guo Y. Chen Y. Zou G. Chen J. Gao X. . (2025). Increased spindle-related brain activation in right middle temporal gyrus during N2 than N3 among healthy sleepers: initial discovery and independent sample replication. NeuroImage 305:120976. doi: 10.1016/j.neuroimage.2024.120976, 39681244

[ref25] Shinto E. Yang S. Shinto A. Kurata J. (2025). A potential role for the middle temporal gyrus in mediating pain rumination in patients with chronic pain. NeuroImage 310:121106. doi: 10.1016/j.neuroimage.2025.121106, 40024554

[ref26] Taft T. H. Triggs J. R. Carlson D. A. Guadagnoli L. Tomasino K. N. Keefer L. . (2018). Validation of the oesophageal hypervigilance and anxiety scale for chronic oesophageal disease. Aliment. Pharmacol. Ther. 47, 1270–1277. doi: 10.1111/apt.14605, 29528128 PMC5897170

[ref27] Thomas K. S. Motivala S. Olmstead R. Irwin M. R. (2011). Sleep depth and fatigue: role of cellular inflammatory activation. Brain Behav. Immun. 25, 53–58. doi: 10.1016/j.bbi.2010.07.245, 20656013 PMC2991567

[ref28] Verdonk C. A. O. Khalsa S. S. (2025). Toward a multidisciplinary neurobiology of interoception and mental health. Curr. Opin. Neurobiol. 94:103084. doi: 10.1016/j.conb.2025.103084, 40616883

[ref29] Wan Y. Sun C. Fan M. Yu H. Xu J. Zhang K. . (2025). Exploring psychological factors and brain alterations in functional anorectal pain patients: insights from multimodal magnetic resonance imaging investigations. Neurogastroenterol. Motility. 37:e15017. doi: 10.1111/nmo.15017, 39901693

[ref30] Wang Z. Wu T. Li J. Lu T. Yu Y. Guan Z. . (2025). Brain-gut-microbiota interactions in sleep disorders. Brain Med. 1, 31–52. doi: 10.61373/bm025i.0128, 37402401

[ref31] Wu Y. Zhuang Y. Qi J. (2020). Explore structural and functional brain changes in insomnia disorder: a PRISMA-compliant whole brain ALE meta-analysis for multimodal MRI. Medicine 99:e19151. doi: 10.1097/MD.0000000000019151, 32243357 PMC7220541

[ref32] Xu J. Zhang J. Li J. Wang H. Chen J. Lyu H. . (2022). Structural and functional trajectories of middle temporal gyrus sub-regions during life span: a potential biomarker of brain development and aging. Front. Aging Neurosci. 14:799260. doi: 10.3389/fnagi.2022.799260, 35572140 PMC9094684

[ref33] Zhang Y. Ji J. Zheng L. Cai M. Sun G. Ma Y. . (2024). Unveiling neuroimmunology profile of immunological non-responders in HIV: a multimodal MRI approach. Front. Immunol. 15:1452532. doi: 10.3389/fimmu.2024.1452532, 39735540 PMC11671397

