# Supplementary material for: Altered cortical neuronal activity in functional esophageal disorders and its associations with chronic insomnia and peripheral inflammation: a resting-state fMRI study
Source: Front Mol Neurosci. 2026 Jul 6;19:1871404. doi: 10.3389/fnmol.2026.1871404 (PMC13381702; doi:10.3389/fnmol.2026.1871404)
Supplement: Supplementary file 1 [file Data_Sheet_1.DOCX]

**Supplementary Materials:**

**Supplementary Table S1. Full correlation matrix between ALFF measures and clinical/inflammatory variables in the FED group (n=19)**

Notes: ALFF, amplitude of low‑frequency fluctuation; MTG, middle temporal gyrus; PSQI, Pittsburgh Sleep Quality Index (components A–G); RDQ, Reflux Disease Questionnaire; EHAS, Esophageal Hypervigilance and Anxiety Scale; NEQOL, Northwestern Esophageal Quality of Life Scale; PHQ‑9, Patient Health Questionnaire‑9; GAD‑7, Generalized Anxiety Disorder‑7; HAMD, Hamilton Depression Rating Scale; HAMA, Hamilton Anxiety Scale.
P values are uncorrected for multiple comparisons. After false discovery rate (FDR) correction (Benjamini–Hochberg, 84 tests), no correlation remained statistically significant (all q ≥ 0.129).
The sample size for most correlations is 19; for variables with missing data (RDQ frequency, RDQ severity, EHAS, NEQOL, PHQ‑9, GAD‑7), n = 18 or 17 as indicated in the original dataset.

| **ALFF measure** | **Variable** | **Spearman’s**  **r** | **Uncorrected**  **P** | **FDR-corrected**  **q** |
| --- | --- | --- | --- | --- |
| **Global ALFF** |  |  |  |  |
|  | PSQI total | -0.064 | 0.796 | 0.9346 |
|  | PSQI‑A (subjective sleep quality) | 0.034 | 0.889 | 0.9408 |
|  | PSQI‑B (sleep latency) | -0.330 | 0.167 | 0.7478 |
|  | PSQI‑C (sleep duration) | 0.195 | 0.424 | 0.8118 |
|  | PSQI‑D (sleep efficiency) | 0.002 | 0.993 | 0.9930 |
|  | PSQI‑E (sleep disturbances) | 0.037 | 0.880 | 0.9408 |
|  | PSQI‑F (use of sleep medication) | 0.249 | 0.305 | 0.7478 |
|  | PSQI‑G (daytime dysfunction) | -0.241 | 0.320 | 0.7478 |
|  | RDQ frequency | -0.096 | 0.705 | 0.9346 |
|  | RDQ severity | -0.075 | 0.775 | 0.9346 |
|  | EHAS | 0.060 | 0.812 | 0.9346 |
|  | NEQOL | 0.087 | 0.732 | 0.9346 |
|  | PHQ‑9 | -0.164 | 0.515 | 0.9010 |
|  | GAD‑7 | -0.260 | 0.298 | 0.7478 |
|  | HAMD | -0.010 | 0.968 | 0.9916 |
|  | HAMA | 0.036 | 0.882 | 0.9408 |
|  | IL‑5 | 0.301 | 0.211 | 0.7478 |
|  | IL‑4 | 0.060 | 0.807 | 0.9346 |
|  | IL‑2 | 0.215 | 0.376 | 0.7642 |
|  | IL‑10 | 0.370 | 0.119 | 0.7478 |
|  | IFN‑α | 0.143 | 0.560 | 0.9346 |
|  | IL‑1β | -0.134 | 0.585 | 0.9346 |
|  | IL‑12p70 | 0.261 | 0.280 | 0.7478 |
|  | **IL‑8** | **0.567** | **0.011** | 0.3370 |
|  | IL‑17A | 0.213 | 0.382 | 0.7642 |
|  | IL‑6 | 0.319 | 0.183 | 0.7478 |
|  | IFN‑γ | 0.245 | 0.313 | 0.7478 |
|  | **TNF‑α** | **0.518** | **0.023** | 0.3856 |
| **L-MTG ALFF** |  |  |  |  |
|  | PSQI total | 0.123 | 0.616 | 0.9346 |
|  | PSQI‑A | 0.064 | 0.796 | 0.9346 |
|  | PSQI‑B | 0.006 | 0.981 | 0.9930 |
|  | PSQI‑C | 0.301 | 0.210 | 0.7478 |
|  | PSQI‑D | 0.287 | 0.233 | 0.7478 |
|  | PSQI‑E | 0.308 | 0.200 | 0.7478 |
|  | PSQI‑F | -0.080 | 0.743 | 0.9346 |
|  | PSQI‑G | -0.325 | 0.174 | 0.7478 |
|  | RDQ frequency | -0.075 | 0.769 | 0.9346 |
|  | RDQ severity | -0.120 | 0.648 | 0.9346 |
|  | EHAS | 0.307 | 0.215 | 0.7478 |
|  | NEQOL | 0.326 | 0.187 | 0.7478 |
|  | PHQ‑9 | 0.219 | 0.382 | 0.7642 |
|  | GAD‑7 | 0.184 | 0.465 | 0.8490 |
|  | HAMD | 0.373 | 0.115 | 0.7478 |
|  | HAMA | 0.289 | 0.230 | 0.7478 |
|  | IL‑5 | 0.262 | 0.279 | 0.7478 |
|  | IL‑4 | -0.152 | 0.534 | 0.9147 |
|  | IL‑2 | 0.227 | 0.349 | 0.7642 |
|  | IL‑10 | 0.221 | 0.363 | 0.7642 |
|  | IFN‑α | -0.069 | 0.778 | 0.9346 |
|  | IL‑1β | -0.186 | 0.446 | 0.8322 |
|  | IL‑12p70 | 0.288 | 0.231 | 0.7478 |
|  | **IL‑8** | **0.675** | **0.002** | 0.1285 |
|  | IL‑17A | 0.286 | 0.235 | 0.7478 |
|  | IL‑6 | 0.437 | 0.061 | 0.7478 |
|  | IFN‑γ | 0.255 | 0.292 | 0.7478 |
|  | **TNF‑α** | **0.563** | **0.012** | 0.3370 |
| **R-MTG ALFF** |  |  |  |  |
|  | PSQI total | 0.109 | 0.657 | 0.9346 |
|  | PSQI‑A | 0.100 | 0.683 | 0.9346 |
|  | PSQI‑B | -0.213 | 0.382 | 0.7642 |
|  | PSQI‑C | 0.094 | 0.703 | 0.9346 |
|  | PSQI‑D | 0.082 | 0.740 | 0.9346 |
|  | PSQI‑E | 0.075 | 0.761 | 0.9346 |
|  | **PSQI‑F** | **0.541** | **0.017** | 0.3535 |
|  | PSQI‑G | -0.077 | 0.754 | 0.9346 |
|  | RDQ frequency | -0.306 | 0.216 | 0.7478 |
|  | RDQ severity | -0.271 | 0.293 | 0.7478 |
|  | EHAS | 0.140 | 0.578 | 0.9346 |
|  | NEQOL | 0.179 | 0.478 | 0.8535 |
|  | PHQ‑9 | 0.200 | 0.425 | 0.8118 |
|  | GAD‑7 | -0.103 | 0.685 | 0.9346 |
|  | HAMD | 0.286 | 0.235 | 0.7478 |
|  | HAMA | 0.249 | 0.303 | 0.7478 |
|  | IL‑5 | 0.277 | 0.250 | 0.7478 |
|  | IL‑4 | -0.246 | 0.310 | 0.7478 |
|  | IL‑2 | 0.029 | 0.907 | 0.9408 |
|  | IL‑10 | 0.083 | 0.736 | 0.9346 |
|  | IFN‑α | -0.410 | 0.081 | 0.7478 |
|  | IL‑1β | -0.106 | 0.666 | 0.9346 |
|  | IL‑12p70 | 0.036 | 0.884 | 0.9408 |
|  | IL‑8 | 0.354 | 0.137 | 0.7478 |
|  | IL‑17A | -0.054 | 0.827 | 0.9389 |
|  | IL‑6 | 0.042 | 0.865 | 0.9408 |
|  | IFN‑γ | 0.031 | 0.901 | 0.9408 |
|  | TNF‑α | 0.320 | 0.181 | 0.7478 |

Note: Bold rows indicate nominally significant correlations (uncorrected P < 0.05). After FDR correction, none of these remained significant (all q ≥ 0.129).

ALFF, amplitude of low-frequency fluctuation; MTG, middle temporal gyrus; PSQI, Pittsburgh Sleep Quality Index; RDQ, Reflux Disease Questionnaire; EHAS, Esophageal Hypervigilance and Anxiety Scale; NEQOL, Northwestern Esophageal Quality of Life Scale; PHQ-9, Patient Health Questionnaire-9; GAD-7, Generalized Anxiety Disorder-7; HAMD, Hamilton Depression Scale; HAMA, Hamilton Anxiety Scale.


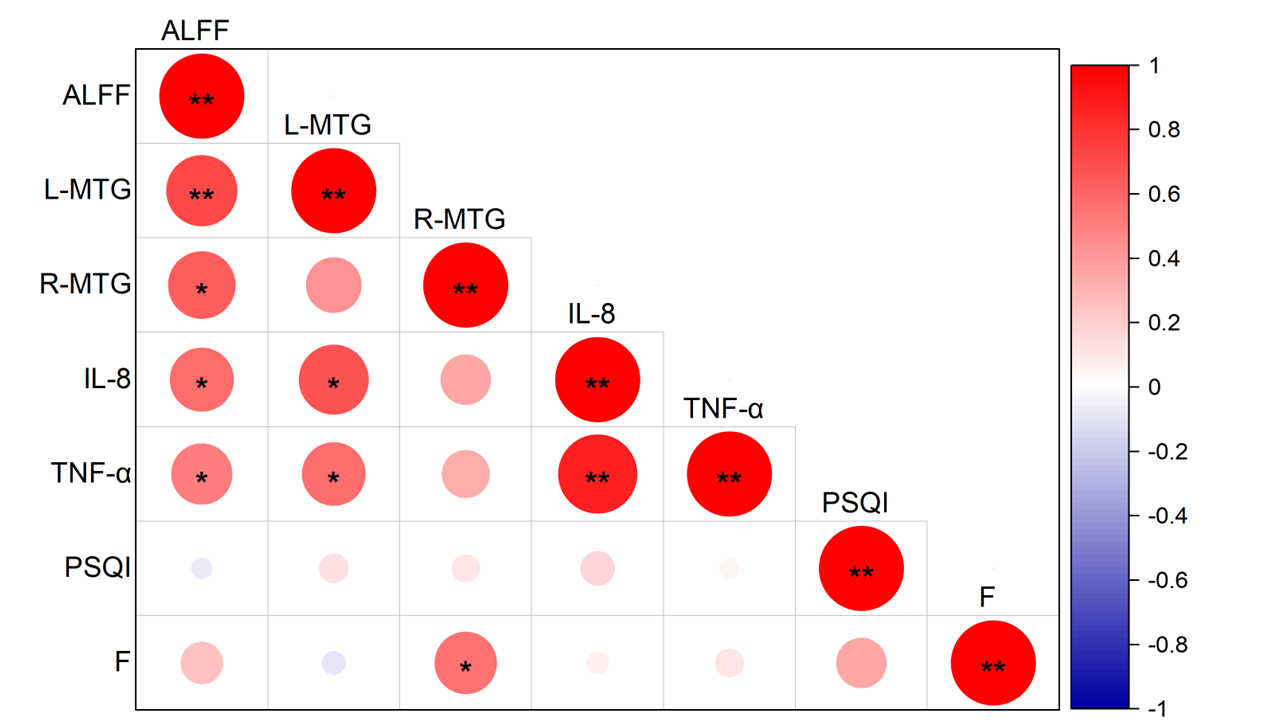


**Figure S1.** Exploratory correlation plot showing nominally significant correlations among ALFF measures, inflammatory markers, and sleep-related variables in the FED group (n = 19). Spearman’s r and uncorrected P values are shown. After FDR correction for 84 comparisons, none of these correlations remained statistically significant (all q ≥ 0.129). This plot is provided for transparency and hypothesis generation only. Asterisks indicate uncorrected P < 0.05 (*) and P < 0.01 (**).

**Supplementary Results**
**Exploratory mediation analysis (Hypothesis‑Generating Only)**

Although no correlation survived FDR correction (all q ≥ 0.129), we performed an exploratory mediation analysis to generate hypotheses for future studies. The model tested **IL‑8** as the predictor, **left MTG ALFF** as the mediator, and **PSQI‑G (daytime dysfunction)** as the outcome, adjusting for age and sex (n = 19). We used PROCESS Model 4 with 5,000 bootstrap resamples.

The results are as follows:

1. Path a (IL‑8 → left MTG ALFF): coefficient = 0.0011, SE = 0.0003, P = 0.0027 (uncorrected).

2. Path b (left MTG ALFF → daytime dysfunction): coefficient = –5.6400, SE = 1.8659, P = 0.0091 (uncorrected).

3. Direct effect (c′, IL‑8 → daytime dysfunction): coefficient = 0.0097, SE = 0.0030, P = 0.0066 (uncorrected).

**4. Indirect effect (via left MTG ALFF):** coefficient = –0.0062, BootSE = 0.0084, **95% bootstrap CI** = –0.0176 to –0.0006.

The indirect effect was nominally significant (bootstrap confidence interval did not include zero). However, **this finding must be interpreted with extreme caution** for the following reasons:

1. The correlation between IL‑8 and left MTG ALFF did not survive FDR correction for multiple comparisons (q = 0.129).

2. The mediation analysis is based on cross‑sectional data; therefore, no causal direction can be inferred.

3. The small sample size (n = 19) leads to unstable effect estimates and potential overfitting.

4. The analysis was not pre‑registered and should be considered **hypothesis generating**only.

No causal claims (e.g., “buffering” or “compensation”) can be made. Independent replication in larger, longitudinal cohorts is required before any firm conclusions can be drawn.


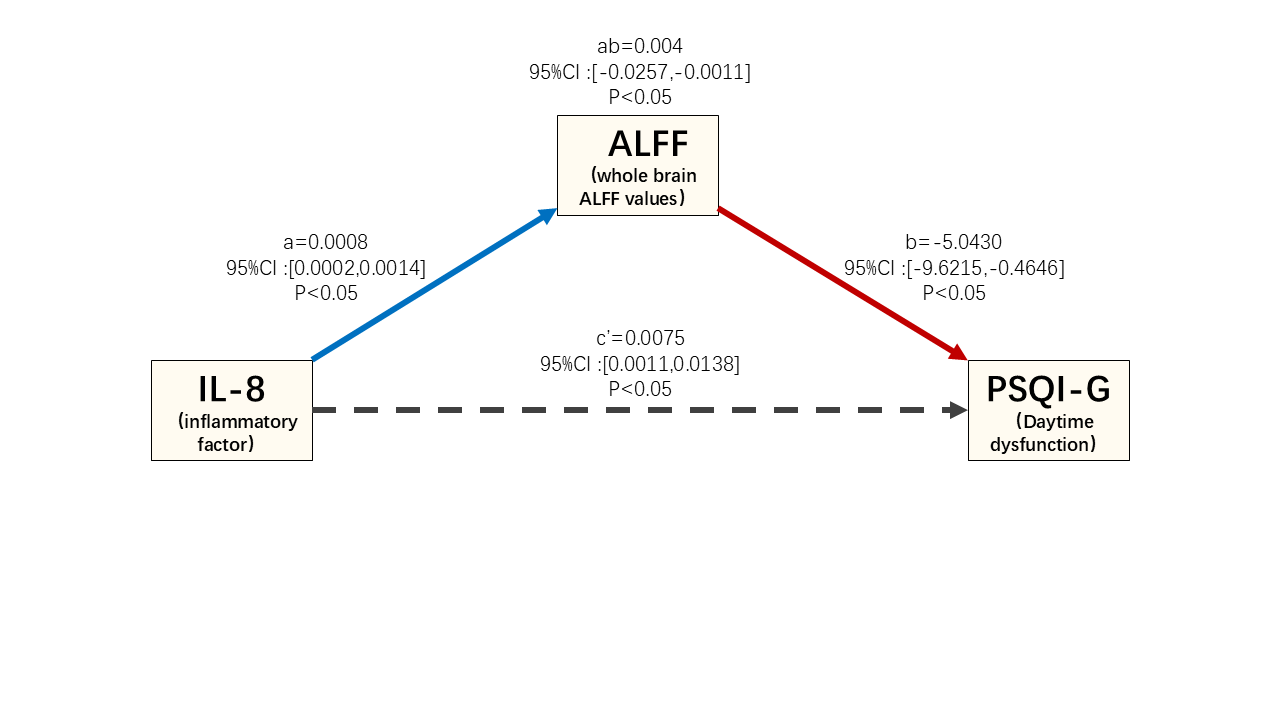


**Figure S2.** Exploratory mediation model of IL‑8, left MTG ALFF, and daytime dysfunction (PSQI‑G) in the FED group (n = 19). Path coefficients are shown with uncorrected P values. The indirect effect (IL‑8 → left MTG ALFF → daytime dysfunction) was nominally significant (95% bootstrap CI: –0.0176 to –0.0006). This analysis is purely exploratory and hypothesis‑generating. Because the correlation between IL‑8 and left MTG ALFF did not survive FDR correction (q = 0.129), and because the data are cross‑sectional, no causal claims can be made. The model adjusted for age and sex. Solid lines indicate nominally significant paths at the uncorrected P < 0.05 level. CI, confidence interval; MTG, middle temporal gyrus; PSQI‑G, Pittsburgh Sleep Quality Index Component G (daytime dysfunction).

**Supplementary Table S2. Full correlation matrix between ALFF measures and sleep quality across all participants (N = 57)**
All 24 Spearman’s rank correlations are shown, including uncorrected P values and FDR‑corrected q values (Benjamini–Hochberg method, 24 tests). Correlations with q < 0.05 are considered significant after correction and are marked as “Yes” in the final column.

| **ALFF**  **measure** | **PSQI**  **component** | **Spearman’s**  **r** | **Uncorrected**  **P** | **FDR‑corrected**  **q** | **Significant**  **(q < 0.05)** |
| --- | --- | --- | --- | --- | --- |
| **Global ALFF** |  |  |  |  |  |
|  | Total score | –0.336 | 0.01057 | 0.036 | Yes |
|  | A (subjective sleep quality) | –0.374 | 0.00419 | 0.020 | Yes |
|  | B (sleep latency) | –0.250 | 0.06033 | 0.072 | No |
|  | C (sleep duration) | –0.295 | 0.02576 | 0.045 | Yes |
|  | D (sleep efficiency) | –0.285 | 0.03159 | 0.046 | Yes |
|  | E (sleep disturbances) | –0.306 | 0.02062 | 0.045 | Yes |
|  | F (use of sleep medication) | –0.005 | 0.96868 | 0.969 | No |
|  | G (daytime dysfunction) | –0.352 | 0.00717 | 0.029 | Yes |
| **Left MTG** |  |  |  |  |  |
|  | Total score | –0.402 | 0.00194 | 0.018 | Yes |
|  | A (subjective sleep quality) | –0.452 | 0.00041 | 0.010 | Yes |
|  | B (sleep latency) | –0.284 | 0.03249 | 0.046 | Yes |
|  | C (sleep duration) | –0.312 | 0.01808 | 0.043 | Yes |
|  | D (sleep efficiency) | –0.381 | 0.00350 | 0.020 | Yes |
|  | E (sleep disturbances) | –0.299 | 0.02384 | 0.045 | Yes |
|  | F (use of sleep medication) | –0.070 | 0.60540 | 0.660 | No |
|  | G (daytime dysfunction) | –0.397 | 0.00222 | 0.018 | Yes |
| **Right MTG** |  |  |  |  |  |
|  | Total score | –0.295 | 0.02594 | 0.045 | Yes |
|  | A (subjective sleep quality) | –0.321 | 0.01495 | 0.040 | Yes |
|  | B (sleep latency) | –0.233 | 0.08172 | 0.093 | No |
|  | C (sleep duration) | –0.268 | 0.04422 | 0.059 | No |
|  | D (sleep efficiency) | –0.329 | 0.01233 | 0.037 | Yes |
|  | E (sleep disturbances) | –0.256 | 0.05411 | 0.068 | No |
|  | F (use of sleep medication) | 0.050 | 0.71408 | 0.745 | No |
|  | G (daytime dysfunction) | –0.290 | 0.02855 | 0.046 | Yes |

Note: MTG, middle temporal gyrus; PSQI, Pittsburgh Sleep Quality Index. FDR correction was performed across 24 tests. q < 0.05 was considered statistically significant. Uncorrected P values are two‑tailed.

**Supplementary fALFF sensitivity analysis**

As a complementary sensitivity analysis, fractional ALFF (fALFF) maps were generated and analyzed using the same statistical framework as the primary ALFF analysis. After applying the same FDR correction criteria, no significant between-group fALFF differences or fALFF–clinical/inflammatory correlations survived correction. Therefore, fALFF results were not interpreted as primary findings. These null findings should be viewed as complementary sensitivity results and require confirmation in larger studies with physiological monitoring.

**Supplementary Table S3. Analytical performance of the 12-plex cytokine assay.**

| Cytokine | Lower limit of detection | Linear range |
| --- | --- | --- |
| IL-1β | ≤2.44 pg/mL | 2.44–10000 pg/mL |
| IL-2 | ≤2.44 pg/mL | 2.44–10000 pg/mL |
| IL-4 | ≤2.44 pg/mL | 2.44–10000 pg/mL |
| IL-5 | ≤2.44 pg/mL | 2.44–10000 pg/mL |
| IL-6 | ≤2.44 pg/mL | 2.44–10000 pg/mL |
| IL-8 | ≤2.44 pg/mL | 2.44–10000 pg/mL |
| IL-10 | ≤2.44 pg/mL | 2.44–10000 pg/mL |
| IL-12p70 | ≤2.44 pg/mL | 2.44–10000 pg/mL |
| IL-17 | ≤2.44 pg/mL | 2.44–10000 pg/mL |
| IFN-α | ≤2.44 pg/mL | 2.44–4000 pg/mL |
| IFN-γ | ≤2.44 pg/mL | 2.44–4000 pg/mL |
| TNF-α | ≤2.44 pg/mL | 2.44–10000 pg/mL |

Note: Cytokine testing was performed uniformly by the hospital clinical laboratory using a 12-plex cytokine detection kit based on flow fluorescence immunoassay on a Beckman Coulter DxFLEX flow cytometer. According to the manufacturer’s instructions, the lower limit of detection for all 12 cytokines was no greater than 2.44 pg/mL, and the intra-assay and inter-assay coefficients of variation were ≤15%.

**Supplementary Table S4. Summary of fALFF sensitivity analyses.**

| **Analysis** | **Correction threshold** | **Result** |
| --- | --- | --- |
| Whole-brain fALFF omnibus group comparison among FED, CID, and HC | Same group-comparison framework and FDR correction as ALFF | No significant clusters survived FDR correction |
| Whole-brain fALFF comparison: HC vs. FED | Same threshold as ALFF | No significant clusters survived FDR correction |
| Whole-brain fALFF comparison: CID vs. FED | Same threshold as ALFF | No significant clusters survived FDR correction |
| FED-group fALFF correlations with clinical/inflammatory variables | FDR correction across tested associations | No correlation survived FDR correction |
| Transdiagnostic fALFF correlations with PSQI measures | FDR correction across tested associations | No correlation survived FDR correction |

Note: fALFF, fractional amplitude of low-frequency fluctuation; FED, functional esophageal disorders; CID, chronic insomnia disorder; HC, healthy controls; PSQI, Pittsburgh Sleep Quality Index. fALFF analyses were performed as complementary sensitivity analyses using the same group-comparison framework and correction thresholds as the primary ALFF analyses.
